# Tandem mass tag-based quantitative proteomic analysis identification of succinylation related proteins in pathogenesis of thoracic aortic aneurysm and aortic dissection

**DOI:** 10.7717/peerj.15258

**Published:** 2023-05-11

**Authors:** Yu Zhang, Hongwei Zhang, Haiyue Wang, Chenhao Wang, Peng Yang, Chen Lu, Yu Liu, Zhenyuan Xu, Yi Xie, Jia Hu

**Affiliations:** 1 Department of Cardiovascular Surgery, West China Hospital, Sichuan University, Chengdu, China; 2 Department of Cardiovascular Surgery, Guang’an Hospital of West China Hospital of Sichuan University, Guang’an, China; 3 Cardiovascular Surgery Research Laboratory, West China Hospital, Sichuan University, Chengdu, China

**Keywords:** Tandem mass tag labelling, Proteomics, Succinylation, OXCT1, Thoracic aortic aneurysm, Thoracic aortic dissection

## Abstract

**Background:**

Thoracic aortic aneurysm and dissection (TAAD) are devastating cardiovascular diseases with a high rate of disability and mortality. Lysine succinylation, a newly found post-translational modification, has been reported to play an important role in cardiovascular diseases. However, how succinylation modification influences TAAD remains obscure.

**Methods:**

Ascending aortic tissues were obtained from patients with thoracic aortic aneurysm (TAA, *n* = 6), thoracic aortic dissection (TAD) with pre-existing aortic aneurysm (*n* = 6), and healthy subjects (*n* = 6). Global lysine succinylation level was analyzed by Western blotting. The differentially expressed proteins (DEPs) were analyzed by tandem mass tag (TMT) labeling and mass spectrometry. Succinylation-related proteins selected from the literature review and AmiGO database were set as a reference inventory for further analysis. Then, the pathological aortic sections were chosen to verify the proteomic results by Western blotting and qRT-PCR.

**Results:**

The level of global lysine succinylation significantly increased in TAA and TAD patients compared with healthy subjects. Of all proteins identified by proteomic analysis, 197 common DEPs were screened both in TAA and TAD group compared with the control group, of which 93 proteins were significantly upregulated while 104 were downregulated. Among these 197 DEPs, OXCT1 overlapped with the succinylation-related proteins and was selected as the target protein involved in thoracic aortic pathogenesis. OXCT1 was further verified by Western blotting and qRT-PCR, and the results showed that OXCT1 in TAA and TAD patients was significantly lower than that in healthy donors (*p* < 0.001), which was consistent with the proteomic results.

**Conclusions:**

OXCT1 represents novel biomarkers for lysine succinylation of TAAD and might be a therapeutic target in the future.

## Introduction

Thoracic aortic aneurysm and dissection (TAAD) are life-threatening conditions, which remain challenge to diagnose and treat. Despite the improvements over the years, overall mortality rates are still up to 50% within the first 48 h (Andreata et al., 2018). The thoracic aorta is consisting of highly dynamic three-layer cell populations and extracellular matrix (ECM). The dysregulation of these components results in vascular smooth muscle cell (VSMC) apoptosis, ECM degradation, and inflammation, leading to progressive dilation, dissection, and rupture when local wall stress continuously increases (Humphrey et al., 2015; Shen et al., 2020). However, no effective pharmacological strategy was found so far to prevent these clinical nightmares from formation and progression. Thus, it is important to quantify mechanisms weakening the aortic wall and to explore potential therapeutic targets for these catastrophic diseases.

Post-translational modification (PTM) is one of the most important regulatory mechanisms of protein function including stability, cellular localization, and activity (Vu, Gevaert & De Smet, 2018). Various PTM types have emerged and are thought to be widely involved in physiological and pathological processes (Liu, Qian & Cao, 2016). Lysine succinylation, a newly identified PTM, metabolically derives succinyl-CoA and modifies protein lysine groups. Succinylation causes a protein charge status from positive (+1) to negative (−1) and introduces a relatively larger structural moiety compared to other PTMs (Zhang et al., 2011). Emerging innovative researches provide evidence on critical role of succinylation modification in numerous biological functions and cardiovascular diseases (Gao et al., 2020; Sadhukhan et al., 2016; Wang et al., 2017). Succinate is an important metabolic molecule of the tricarboxylic acid (TCA) cycle, and has been confirmed to accumulate in TAAD patients (Cui et al., 2021). After entering the cells, succinate can be converted back to succinyl-CoA by succinyl-CoA synthetase in the reversal of TCA cycle. Succinyl-CoA, as a potential cofactor of lysine succinylation, is an important metabolic intermediate in several metabolic processes including the TCA cycle, and could enhance lysine succinylation (Zhang et al., 2011). However, the contribution of succinylation to TAAD and its clinical relevance remains unclear.

To date, quantitative proteomics technology has dramatically improved as a precise method to identify differentially expressed proteins (DEPs) for predicting the underlying mechanisms or therapeutic biomarkers (Geyer et al., 2017; Tyagi & Bhide, 2021). In this study, tandem mass tag (TMT)-based quantitative proteomics technology is used to investigate DEPs in patients with TAAD, further identify key proteins related to succinylation and elucidate their functions in TAAD.

## Materials and Methods

### Tissue samples

The ascending aortic tissues from six thoracic aortic aneurysm (TAA) patients and six thoracic aortic dissection (TAD) patients with pre-existing aneurysms were extracted during open surgical repair. Computed tomography angiography (CTA) was performed in all patients to confirm the diagnosis. Ascending aortas from six organ donors served as controls. All patients had no family history of Marfan syndrome or other connective tissue diseases. More detailed clinical descriptions are summarized in Table 1.

**Table 1:** Baseline characteristics of the patients enrolled in the study.

	Control	TAA	TAD
No.	6	6	6
Gender, male (%)	6 (100%)	6 (100%)	6 (100%)
Age, y	44.7 ± 6.3	56.5 ± 8.2	54.2 ± 6.7
BMI (kg/m^2^)	23.3 ± 3.3	23.4 ± 2.7	24.8 ± 2.3
Hypertension (%)	2 (33.3%)	4 (66.7%)	4 (66.7%)
Smoking (%)	1 (16.7%)	2 (33.3%)	1 (16.7%)
Ascending aortic diameter (mm)	N/A	61.0 ± 6.2	56.2 ± 4.4

**Note:**

TAA, thoracic aortic aneurysm; TAD, thoracic aortic dissection; BMI, body mass index.

All tissues were cut into approximately 1.0 × 1.0 cm^2^ in size. Specimens were immediately frozen in liquid nitrogen and then stored in −80 °C refrigerator. The study was approved by the ethics committee of West China Hospital, Sichuan University (Sichuan, China; No. 38/2020). Written informed consent was obtained from all participants or their legal surrogates before enrolment.

### Protein extraction and tryptic digestion

The tissues were carefully removed from the frozen tubes and diced into 50 mg pieces. Ten times extraction buffer was added to extract the proteins. To get rid of the tissue remnant, the extracted samples were spun.

Tissues were ground individually and lysed with ice-cold lysis buffer containing 100 mM NH_4_HCO_3_(pH 8), 6M Urea, and 0.2% SDS before ultrasonication on ice and centrifuged at 12,000×g for 15 min at 4 °C. For PTM detection, 3 μM trichostatin A (HY-15144; MedChemExpress, Monmouth Junction, NJ, USA), 50 mM nicotinamide (HY-B0150; MedChemExpress, Monmouth Junction, NJ, USA) and 1% phosphatase inhibitor (P1261; Solarbio, Beijing, China) were also added. BCA kit (Solarbio, Beijing, China) was used to measure the protein concentration of the supernatant. The reduction was performed with 10 mM DL-dithiothreitol for 1 h at 56 °C. Subsequently, sufficient iodoacetamide was added for 1 h alkylation in the dark at room temperature. Then samples were incubated at −20 °C for 2 h after thoroughly mixed with four times the volume of precooled acetone. Following centrifugation, the precipitation from the samples was collected, followed by dissolved in dissolution buffer (0.1M triethylammonium bicarbonate and 6M urea). Trypsin was added at 1:50 and 1:100 trypsin-to-protein mass ratios for initial overnight and second 4 h digestion, respectively. TMT 10-plex Label Reagant (Thermo Fisher Scientific, Waltham, MA, USA) was then added for 2 h at room temperature and pooled, desalted and lyophilized. Table S1 shows the TMT labeling information.

### MS/MS analysis of peptides

Solvent A (2% acetonitrile, pH 10.0) was used to resuspend the peptides, and separation was performed in EASY-nLC 1200 UPLC system (Thermo Fisher Scientific, Waltham, MA, USA). The liquid phase gradient follows: 94% solvent A and 6% solvent B within 2 min; 83% solvent A and 17% solvent B within 80 min; 60% solvent A and 40% solvent B within 2 min; 45% solvent A and 55% solvent B within 21 min; 100% solvent B within 5 min (solvent B: 98% acetonitrile, pH 10.0). After separation, peptides were injected into Q Exactive HF-X mass spectromer (Thermo Fisher Scientific, Waltham, MA, USA) with the following settings: spray voltage of 2.3 kV; ion transport capillary temperature of 320 °C; 350−1,500 m/z of primary scanning range; 60,000 resolution of primary MS (at m/z 200); 15,000 resolution of secondary scanning range (at m/z 200); 5 × 10^4^ target value of automatic gain control (AGC); 1.9 × 10^5^ of intensity threshold; 45 ms of maximum ion injection time and 20 s of dynamic exclusion parameter.

### Data analysis

The acquired MS/MS data were analyzed against a Uniprot *Homo sapiens* database (2020_1_8) by Protein Discoverer (PD 2.2; Thermo Fisher Scientific, Waltham, MA, USA). Mass tolerance was set as 10 ppm and fragment mass tolerance was 0.02 Da. Carbamidomethyl on Cys was specified as fixed modifications, while oxidation Met (M) and N-terminus acetylation were set as variable modifications. TMT quantification was performed using Reporter Quantification (TMT 10-plex). False discovery rates were set as no more than 1.0% for identification of protein. Proteins containing similar peptides that indistinguishable in MS/MS analysis were identified as the same group. Mann-Whitney Test was used to statistically examine the protein quantitation results. Proteins were classified as DEPs if their quantification significantly differed between groups (fold change >1.2 or <0.83, *p* < 0.05).

### Selection of succinylation-related proteins

We compiled a list of proteins related to succinylation according to previous published data and gene ontology (GO) terms on the AmiGO database (amigo.geneontology.org) (Bai et al., 2022; Carbon et al., 2009). The terms selected were protein succinylation, succinyl-CoA metabolic process, succinate-CoA ligase complex, succinyl-CoA:oxalate CoA-transferase, succinyl-CoA:(R)-benzylsuccinate CoA-transferase activity, succinyl-CoA binding, succinate-CoA ligase activity, succinyl-CoA hydrolase activity, succinyl-CoA:(R)-citramalate CoA-transferase activity, succinyl-CoA:3-oxo-acid CoA-transferase activity, succinate metabolic process, succinate dehydrogenase activity and succinate transport. Table S2 lists all the annotated terms and their respective GO identifications (GO ID).

### Biological process enrichment analysis

Gene ontology (GO) enrichment was analyzed using the interproscan-5 program against the non-redundant protein database (including Pfam, PRINTS, ProDom, SMART, ProSiteProfiles, PANTHER). Kyoto encyclopedia of genes and genomes (KEGG) analysis was performed to analyze the pathway using Fisher’s extract test. The GO and KEGG enrichment analysis was conducted using the enrichment pipeline.

### Western blotting assay

Human aortic tissues were lysed with RIPA buffer containing 3 mM trichostatin A, 50 mM nicotinamide, and 1% phosphatase inhibitor. BCA kit (Solarbio, Beijing, China) was used to measure the protein concentration of the supernatant. The lysates (30 μg) were separated by 4–20% MOPS-PAGE (ACE Biotechnology, Nanjing, China) and transferred to nitrocellulose filter membranes (Merck Millipore, Cork, IRL, USA). The membranes were blocked using 5% (w/v) nonfat dry milk in TBST Buffer, and incubated with primary antibodies against succinyllysine (PTM-401; PTM Biolabs Inc., Hangzhou, China), acetyllysine (PTM-105RM; PTM Biolabs Inc., Hangzhou, China), malonyllysine (PTM-902; PTM Biolabs Inc., Hangzhou, China), ubiquitin (PTM-1106RM; PTM Biolabs Inc., Hangzhou, China), L-lactyl lysine (PTM-1401RM; PTM Biolabs Inc., Hangzhou, China) and OXCT1 (12175-1-AP; Proteintech, Wuhan, China). Peroxidase-labeled anti-rabbit or anti-mouse IgG (ZSGB-Bio, Beijing, China) was used to probe the bound antibodies. The protein bands were visualized using enhanced chemiluminescence (Bio-Rad, Hercules, CA, USA).

### Quantitative real-time PCR (qRT-PCR)

Total RNA was extracted from the harvested aortic samples with TRIzol Reagent (Invitrogen, Waltham, MA, USA). Reverse transcription was carried out by PrimerScript RT reagent kit (Takara, Shiga, Japan) using 1 µg of total RNA, followed by real-time PCR analysis with SYBR Premix Ex Taq TM II (Takara, Kusatsu, Japan). The GAPDH gene was used as the endogenous control. Primer sequences were as follows: OXCT1 (Forward primer: 5′-AAGCCAAGAGAGGTGAGGGA-3′; Reverse primer: 5′-CACTGTGGTTTCTGCAGCTT-3′); GAPDH (Forward primer: 5′-GGAGCGAGATCCCTCCAAAAT-3′; Reverse primer: 5′-GGCTGTTGTCATACTTCTCATGG-3′). The relative expression of OXCT1 to GAPDH for each sample was calculated by ΔΔ Ct and expressed as 2^–ΔΔ Ct^.

### Statistical analysis

Bioinformatic analysis were performed in R software (version 4.1.1). Continuous variables were shown as mean ± standard deviation, and categorical variables were summarized as absolute numbers and percentages. Continuous variables were assessed for normality. Student’s t test was performed to make comparisons between two groups of normally distributed data, and one-way analysis of variance, followed by Tukey’s *post hoc* test, was used for multiple comparisons. The Mann Whitney test was performed for non-normal or non-parametric analyses. *P* value less than 0.05 was considered statistically significant. Statistical analyses were performed with SPSS version 24.0 (SPSS Inc., Chicago, IL, USA).

## Results

### Protein lysine succinylation is high in thoracic aortic aneurysm and thoracic aortic dissection tissues

**Figure 1:**
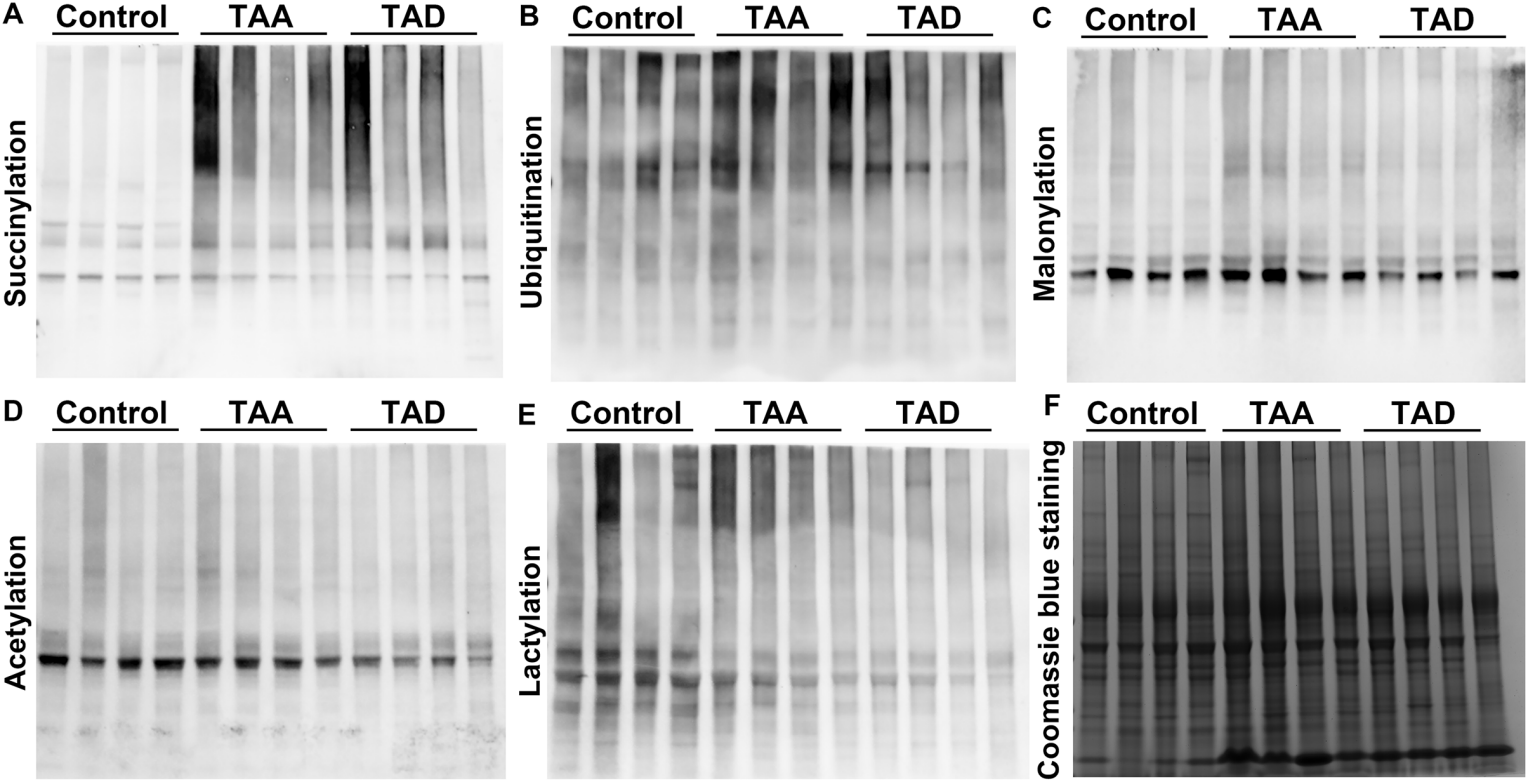
Protein lysine succinylation significantly increased in TAA and TAD tissues. Western blotting results of lysine succinylation (A), ubiquitination (B), malonylation (C), acetylation (D), and lactylation (E). (F) Coomassie brilliant blue staining. TAA, thoracic aortic aneurysm. TAD, thoracic aortic dissection.

We first investigated the PTM levels including succinylation, ubiquitination, malonylation, acetylation, and lactylation in different tissues from TAA patients, TAD patients, and healthy subjects. Western blotting analysis for succinyllysine demonstrated that the levels of succinylation significantly increased in TAA and TAD patients, while others did not change remarkably (Fig. 1).

### Quantitative proteomics among thoracic aortic aneurysm, thoracic aortic dissection, and normal thoracic arteries

**Figure 2:**
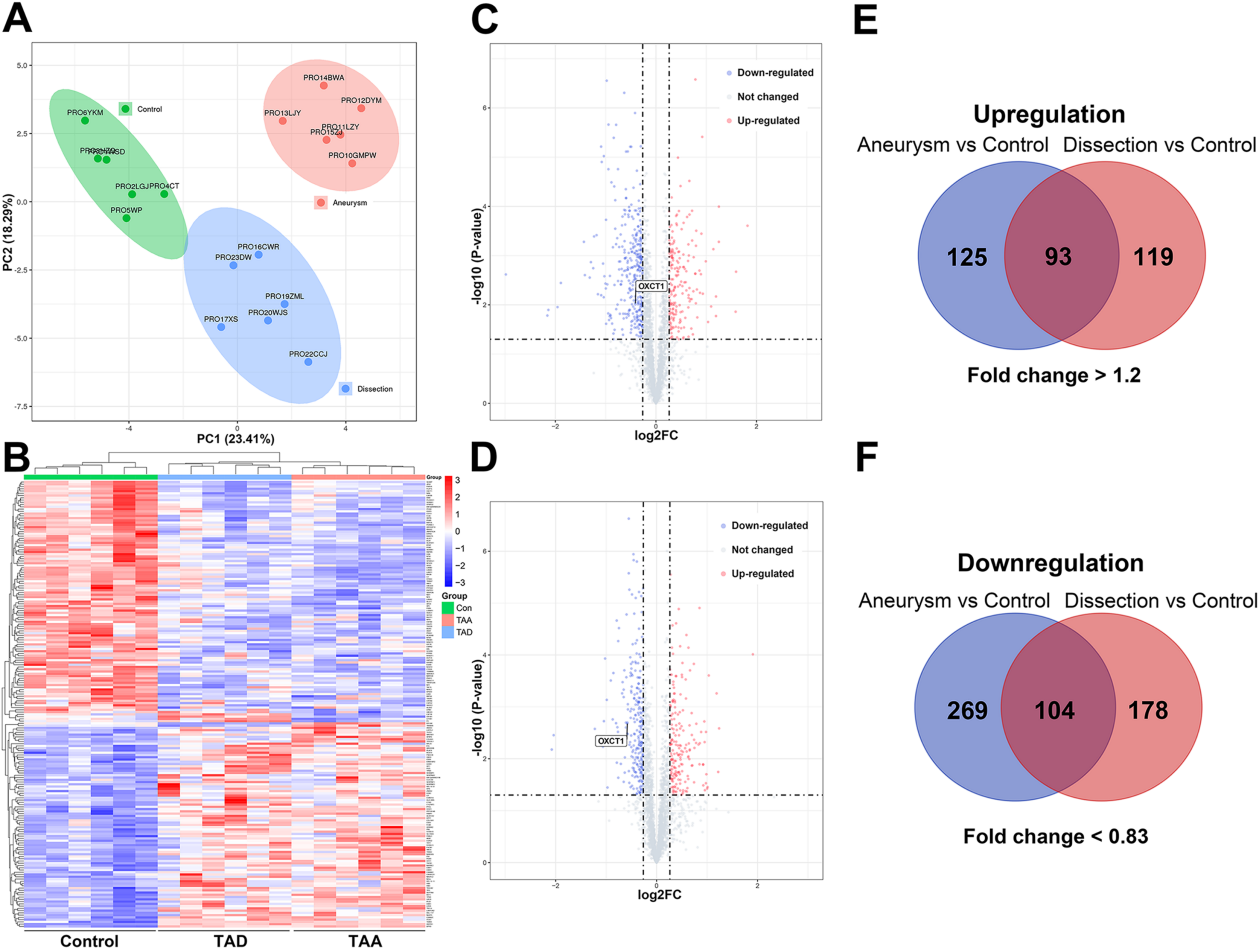
Proteomic analysis of the aortic samples. (A) Principal component analysis (PCA) of proteome data. (B) Expression heat map of all the proteins from TAA patients, TAD patients and healthy subjects. (C) Volcano plots of proteins in ascending aortic tissues from TAA patients and healthy subjects. (D) Volcano plots of proteins in ascending aortic tissues from TAD patients and healthy subjects. (E and F) Overlap evaluation of DEPs in three groups. TAA, thoracic aortic aneurysm. TAD, thoracic aortic dissection. DEPs, differentially expressed proteins.

Principal component analysis (PCA) clustergram was performed and protein profiles of all three groups have consistent clustering as shown in PCA analysis (Fig. 2A). The proteomics profiles of the TAA and TAD groups were distinctively different from those of the control group (Fig. 2B). A total of 3,293 proteins were identified from aortic aneurysm patients and healthy subjects and 3,267 proteins were identified from aortic dissection patients and healthy subjects. The differential proteins were screened with a *p* value < 0.05 by two-sided, unpaired t-test and a fold change >1.2 or <0.83 compared with controls.

Compared to the control group, 591 DEPs were identified in the TAA group, among which 218 proteins were upregulated and 373 proteins were downregulated; a total of 494 DEPs were identified in the TAD group, among which 212 proteins were upregulated and 282 proteins were downregulated. Among these DEPs, 197 proteins (93 upregulated and 104 downregulated) were commonly identified in TAA and TAD groups (Figs. 2C–2F). The details of these DEPs are presented in Tables S3 and S4.

### Functional enrichment analysis of commonly expressed DEPs

**Figure 3:**
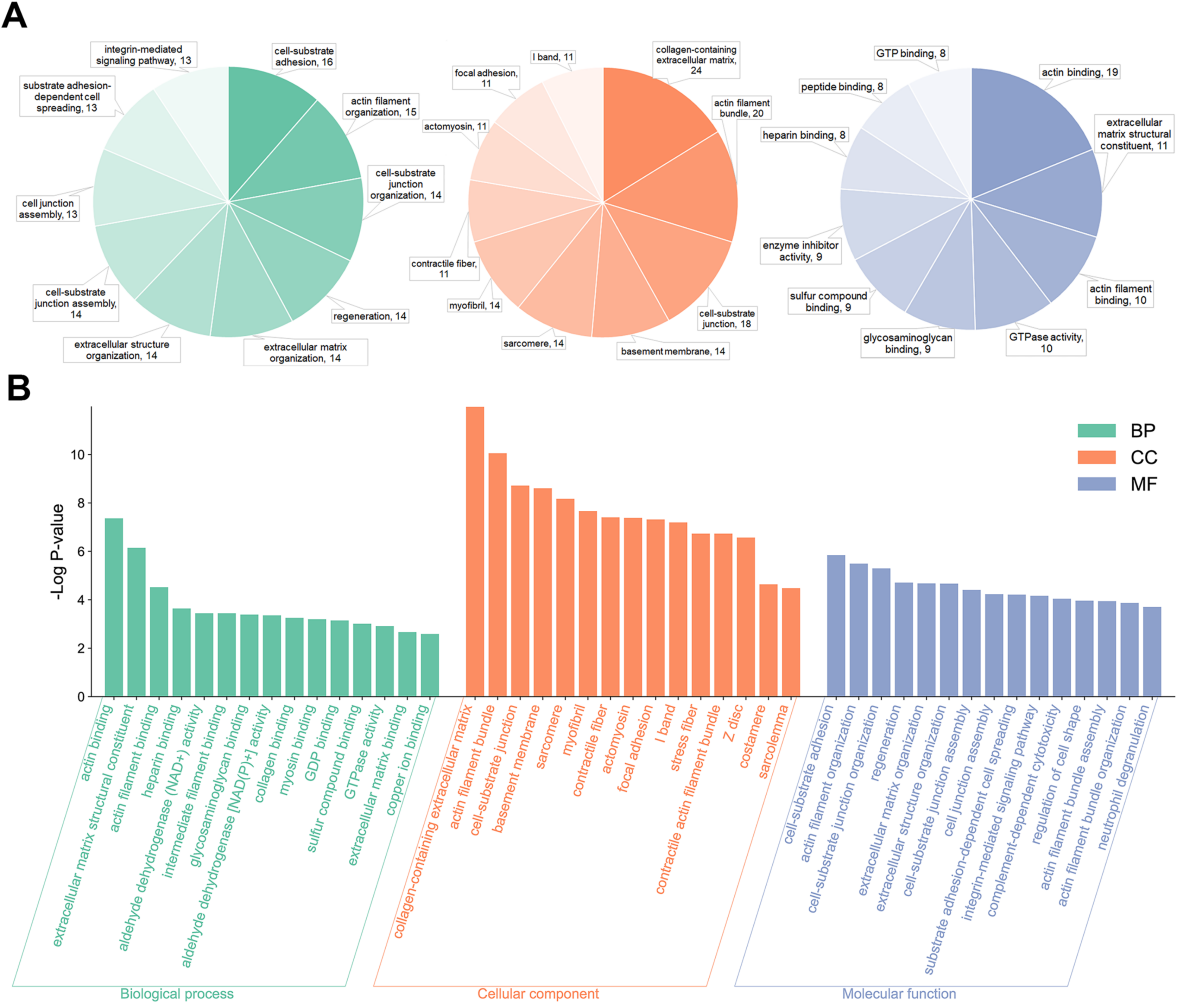
The Gene Ontology (GO) enrichment of differentially expressed proteins (DEPs). (A) Pie chart of the GO enrichment analysis results sorted by protein counts (top 10). (B) Columnar section of the GO enrichment analysis results sorted by −log *P*-value (top 15).

**Figure 4:**
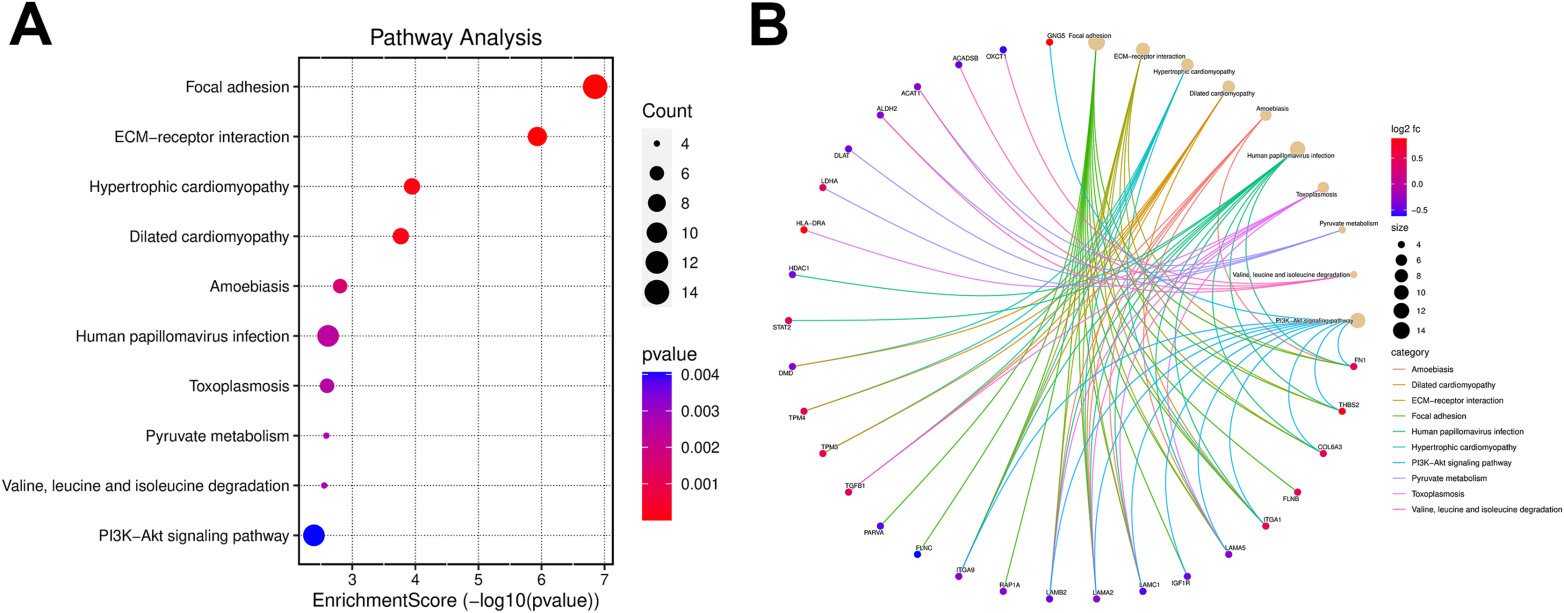
The Kyoto Encyclopedia of Genes and Genomes (KEGG) pathway enrichment analysis for differentially expressed proteins (DEPs). (A) The bubble chart of the KEGG pathway enrichment analysis results. (B) The cnetplot of the KEGG pathway enrichment analysis results.

Gene ontology analysis was performed to further clarify the underlying mechanism by biological process, cell components, and molecular function. According to the GO results, most DEPs were located in the extracellular region and participated in several functions, such as actin binding, cell adhesion molecule binding, and extracellular matrix structural constituent (Fig. 3). KEGG pathway enrichment-based clustering analysis was used to further identify the potential pathways. Most of the DEPs were mapped on “focal adhesion”, “PI3K-Akt signaling pathway” and “ECM-receptor interaction” pathways (Fig. 4).

### Identification of succinylation-related proteins

**Figure 5:**
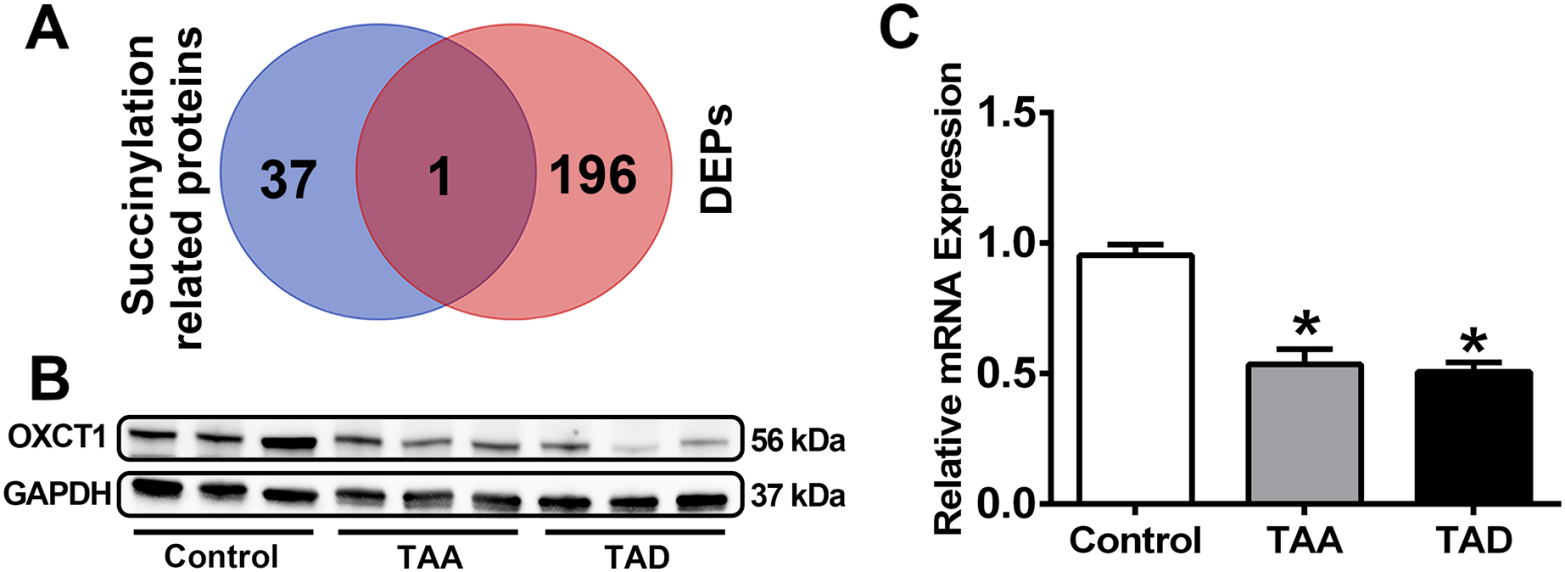
Identification and verification of OXCT1. (A) Venn diagram of succinylation-related DEPs. (B and C) OXCT1 expression in pathological sections of TAA patients, TAD patients and healthy controls by Western blotting and qRT-PCR. (*P < 0.001 *vs* healthy control group) TAA, thoracic aortic aneurysm. TAD, thoracic aortic dissection. DEPs, differentially expressed proteins.

Initially, a list of succinylation-related proteins was generated, as described in detail in the methods. This yielded a list of 38 related proteins (Table 2), including 10 enzymes related to succinylation modification (SIRT1, SIRT2, SIRT3, SIRT4, SIRT5, SIRT6, SIRT7, CPT1A, KAT5, and GLYATL1), 19 succinate metabolizing enzymes (SDHA, SDHB, SDHC, SDHD, SUCLA2, SUCLG1, SUCLG2, CREBBP, OXCT1, MEAF6, SSDH, SLC13A2, SLC13A3, SLC13A5, SLC16A1, SLC25A10, SLC25A11, SLC25A14, and SLC25A30) and 13 succinyl-CoA metabolizing enzymes (DLST, MMUT, ACOT4, ACOT8, NUDT7, NUDT8, NUDT19, SUCLA2, SUCLG1, SUCLG2, OGDH, OXCT1, and OXCT2). Among these succinylation-related proteins, one overlapped with the DEPs obtained as described above, namely, OXCT1 (Fig. 5A).

**Table 2:** Succinylation-related proteins from literature review and AmiGO database.

Protein symbol	Protein name
Category: succinate metabolizing enzymes	
SDHA	Succinate dehydrogenase complex flavoprotein subunit A
SDHB	Succinate dehydrogenase complex iron sulfur subunit B
SDHC	Succinate dehydrogenase complex subunit C
SDHD	Succinate dehydrogenase complex subunit D
SUCLA2	Succinate-CoA ligase ADP-forming subunit beta
SUCLG1	Succinate-CoA ligase GDP/ADP-forming subunit alpha
SUCLG2	Succinate-CoA ligase GDP-forming subunit beta
CREBBP	CREB binding protein
OXCT1	3-oxoacid CoA-transferase 1
MEAF6	MYST/Esa1 associated factor 6
SSDH	Succinate-semialdehyde dehydrogenase
SLC13A2	Solute carrier family 13 member 2
SLC13A3	Solute carrier family 13 member 3
SLC13A5	Solute carrier family 13 member 5
SLC16A1	Solute carrier family 16 member 1
SLC25A10	Solute carrier family 25 member 10
SLC25A11	Solute carrier family 25 member 11
SLC25A14	Solute carrier family 25 member 14
SLC25A30	Solute carrier family 25 member 30
Category: succinyl-CoA metabolizing enzymes	
DLST	Dihydrolipoamide S-succinyltransferase
MMUT	Methylmalonyl-CoA mutase
ACOT4	Acyl-CoA thioesterase 4
ACOT8	Acyl-CoA thioesterase 8
NUDT7	Nudix hydrolase 7
NUDT8	Nudix hydrolase 8
NUDT19	Nudix hydrolase 19
SUCLA2	Succinate-CoA ligase ADP-forming subunit beta
SUCLG1	Succinate-CoA ligase GDP/ADP-forming subunit alpha
SUCLG2	Succinate-CoA ligase GDP-forming subunit beta
OGDH	Oxoglutarate dehydrogenase
OXCT1	3-oxoacid CoA-transferase 1
OXCT2	3-oxoacid CoA-transferase 2
Category: succinylation modification related enzymes	
SIRT1	Sirtuin 1
SIRT2	Sirtuin 2
SIRT3	Sirtuin 3
SIRT4	Sirtuin 4
SIRT5	Sirtuin 5
SIRT6	Sirtuin 6
SIRT7	Sirtuin 7
CPT1A	Carnitine palmitoyltransferase 1A
KAT5	Lysine acetyltransferase 5
GLYATL1	Glycine-N-acyltransferase like 1

### Evaluation of OXCT1 by Western blotting and qRT-PCR

The expression levels of OXCT1 in pathological sections were further assessed by Western blotting and qRT-PCR (Figs. 5B and 5C, Fig. S1). The results showed significantly decreased levels of OXCT1 in TAA and TAD patients relative to healthy controls (*p* < 0.001), confirming the differential expression patterns determined in proteomic analysis.

## Discussion

In the current study, high levels of succinylation were found to occur in patients with TAAD. Further proteomics analysis found that one succinylation-related protein, OXCT1, was significantly downregulated in both TAA and TAD patients, suggesting that OXCT1 may play an important role in succinylation modification of TAAD. Moreover, this study reports lysine succinylation in TAAD for the first time and warrants exploring strategies for the disease progression. However, the mechanisms between succinylation modification and the development of TAAD remain to be studied further.

TAAD are the most devastating aortic emergencies with a high prevalence and cause significant morbidity and mortality. Especially for untreated ascending aortic dissection, earlier surgical intervention is required with an increased mortality rate of 1–2% per hour (Evangelista et al., 2018; Xing et al., 2020). Several medical therapies (β-blockers, angiotensin-II receptor blockers, angiotensin-converting enzyme inhibitors, *etc*.) have been used to treat TAAD. However, to date, no drugs have been proven to effectively prevent the development and progression of these diseases (Lindeman & Matsumura, 2019; Milewicz & Ramirez, 2019). Detailed mechanisms weakening the aortic wall still need to be clarified.

Previous study demonstrated increased plasma succinate levels in patients with TAAD, which caused mitochondrial dysfunction and subsequent accumulation of reactive oxygen species, thereby exacerbating aortic disease progression (Cui et al., 2021). Succinate, a byproduct of the TCA cycle, plays a critical role in energy production in mitochondria. Accumulation of succinate has also been shown to be an important source of succinyl-CoA, which serves as a succinyl donor to lysine and increases succinylation modification(Mills & O’Neill, 2014; Yang & Gibson, 2019). In this study, the succinylation level in the TAA and TAD groups is significantly higher than in the control group, suggesting that succinylation may play an important role in TAAD formation. However, the associated mechanisms of succinylation in TAAD are yet to be explored.

To define potential proteins related to lysine succinylation, we first performed TMT-based quantitative proteomic analysis to obtain protein expression profiles of ascending aortic tissues from normal controls and from patients with TAA and TAD. A total of 93 proteins were found to be commonly upregulated while 104 were commonly downregulated in ascending aortic tissues of TAA and TAD patients compared with those of healthy donors. Most of these DEPs were located in the extracellular region part and their biological functions mainly focused on cellular adhesion and extracellular matrix structural constituent, which was consistent with previous researches (Kjellqvist et al., 2013; Wang et al., 2020; Xing et al., 2020). Then, 38 proteins selected from previous reports and the AmiGO database were set as a reference inventory for succinylation-related DEPs. Notably, OXCT1 was significantly downregulated in TAA and TAD patients, which was confirmed by qRT-PCR.

OXCT1, also called succinyl-CoA:3-ketoacid CoA transferase (SCOT), catalyzes the conversion of succinyl-CoA to acetoacetyl-CoA, which was further metabolized for energy production (Fukao et al., 2014; Grünert et al., 2021). Reduction of OXCT1 was associated with increased succinyl-CoA levels *in vitro* (Dong et al., 2022). Concentrations of succinyl-CoA can regulate succinylation through both enzymatic and non-enzymatic mechanisms. Mixing succinyl-CoA with several proteins increases succinylation in a PH and dose-dependent manner. Altering succinyl CoA metabolism through genetically mutation of specific enzymes in the tricarboxylic acid (TCA) cycle also has a global effect on succinylation levels (Weinert et al., 2013). Succinylation is closely related to major metabolic pathways including TCA cycle, oxidative phosphorylation, ketogenesis, lipid and amino acid metabolism, all of which are responsible for energy transfer or ATP synthesis (Sadhukhan et al., 2016; Yang et al., 2021). Increased succinylation levels could results in impaired mitochondrial enzyme activity and respiration, thus affecting energy homeostasis (Wang et al., 2019). As ascending aorta dilate, the aneurysms exhibited significantly increased energy loss, which is related to aortic size and elastin and collagen composition imbalance (Chung et al., 2014). Moreover, desuccinylation of critical enzymes in TCA cycle can maintain cellular NADPH homeostasis and the ability to cope with oxidative stress (Zhou et al., 2016). Oxidative stress has also been reported to increase vascular wall shear stress by regulating elastic fiber production, thus leading to aortic dissection (Xia et al., 2020). Notably, oxidative stress could enhance VSMC apoptosis, leading to impaired aortic wall remodeling and proteolysis, which may accelerate tissue injury (Wu et al., 2013). These results indicate that OXCT1-induced succinylation modification may be associated with the development and progression of TAAD.

As a key enzyme for ketone body catabolism, the activity and expression of OXCT1 also indicate the level of ketone body utilization (Zeng et al., 2019). Hepatic tissue predominantly produces ketone bodies, which are further transported to extrahepatic tissues for terminal oxidation (Puchalska & Crawford, 2017). Impaired ketone body metabolism due to low levels and deficiency of OXCT1 could result in ketolytic disorders (Zhang & Xie, 2017). Thereinto, β-hydroxybutyrate accounts for the majority of ketone bodies in circulation. A previous study showed that β-hydroxybutyrate selectively reduced TGFβ-dependent responses of ECM genes like Fn1 and limits collagen accumulation (Lecoutre et al., 2022). The impaired integrity of ECM components could destroy aortic structure and function (Andreata et al., 2018; Shen et al., 2020). Similarly, our study found that TGFβ and several ECM-related proteins including Fn1 and collagen were significantly altered.

Additionally, OXCT1 has been suggested to play an important role in regulating autophagy. Knockdown of OXCT1 could promote cell autophagy by elevating AMPK phosphorylation (Huang et al., 2016). Autophagy is a well-conserved and self-degradation process that maintains cellular homeostasis. Accumulating evidence has revealed that autophagy is closely related to TAAD (Fang et al., 2022). Previous studies have shown that autophagy is activated during TAAD formation, and that enhanced autophagy can lead to the autophagic death of VSMCs (Li et al., 2018; Wang et al., 2016). However, there is still debate on the effects of autophagy on TAAD, as other results have also revealed that autophagy deficiency by specifically deletion of VSMC autophagy genes promoted aneurysm formation and aortic rupture (Clément et al., 2019; Irace et al., 2021; Ramadan et al., 2018). Thus, further research is required to validate the role of OXCT1 in VSMC autophagy.

Taken together, the mechanism of OXCT1 in TAAD is likely to be complex and multifactorial, involving the regulation of lysine succinylation, oxidative stress, ketone body metabolism, autophagy, and possibly other factors as well. However, experiments in animals and humans need to be further explored.

## Conclusions

In conclusion, proteomic and succinylation levels were significantly altered in TAA and TAD patients. These results indicate that changes in protein succinylation may play an important role in the development and progression of TAAD. Targeting protein succinylation may open new horizons in therapeutic interventions for TAAD and OXCT1 could be a potential therapeutic target. These hypotheses, however, need to be confirmed by further experiments with larger sample size.

## Supplemental Information

10.7717/peerj.15258/supp-1 Supplemental Information 1 Western blotting validation for OXCT1 of another 9 samples.

10.7717/peerj.15258/supp-2 Supplemental Information 2 TMT reagent labels.

10.7717/peerj.15258/supp-3 Supplemental Information 3 Gene ontology terms related to protein succinylation.

10.7717/peerj.15258/supp-4 Supplemental Information 4 Upregulated differentially expressed proteins in TAA and TAD patients compared with healthy controls.

10.7717/peerj.15258/supp-5 Supplemental Information 5 Downregulated differentially expressed proteins in TAA and TAD patients compared with healthy controls.

10.7717/peerj.15258/supp-6 Supplemental Information 6 Raw data for baseline characteristic and qRT-PCR.

10.7717/peerj.15258/supp-7 Supplemental Information 7 Raw data for proteomics. All proteins identified by TMT proteomics. These data were used for statistical analysis to identify differentially expressed proteins.

10.7717/peerj.15258/supp-8 Supplemental Information 8 Original western blots.

## Additional Information and Declarations

### Data Availability

The following information was supplied regarding data availability: The raw data of proteomics are available in the Supplemental File.
