# Supplementary material for: Tandem mass tag-based quantitative proteomic analysis identification of succinylation related proteins in pathogenesis of thoracic aortic aneurysm and aortic dissection
Source: PeerJ. 2023 May 11;11:e15258. doi: 10.7717/peerj.15258 (PMC10183161; doi:10.7717/peerj.15258)
Supplement: Supplemental Information 2 [file peerj-11-15258-s002.docx]

**Table S1**. TMT reagent labels.

| **Control samples**  **Run 1** | **TAA samples**  **Run 2** | **TAD samples**  **Run 3** |
| --- | --- | --- |
| Mix/F1:126 | Mix/F1:126 | Mix/F1:126 |
| Control 1/F1:127N | TAA 1/F1:127N | TAD 1/F1:128C |
| Control 2/F1:127C | TAA 2/F1:127C | TAD 2/F1:129N |
| Control 3/F1:128N | TAA 3/F1:128N | TAD 3/F1:129C |
| Control 4/F1:128C | TAA 4/F1:130N | TAD 4/F1:130N |
| Control 5/F1:129N | TAA 5/F1:130C | TAD 5/F1:130C |
| Control 6/F1:129C | TAA 6/F1:131 | TAD 6/F1:131 |
| Note: An equal amount of protein of each sample were mixed into one as a control (Mix). 126, 127N, 127C, 128N, 128C, 129N, 129C, 130N,130C, and 131 represent TMT Reagent Labels. | | |
