# Supplementary material for: Tandem mass tag-based quantitative proteomic analysis identification of succinylation related proteins in pathogenesis of thoracic aortic aneurysm and aortic dissection
Source: PeerJ. 2023 May 11;11:e15258. doi: 10.7717/peerj.15258 (PMC10183161; doi:10.7717/peerj.15258)
Supplement: Supplemental Information 3 [file peerj-11-15258-s003.docx]

**Table S2**. Gene ontology terms related to protein succinylation.

| **Gene Ontology Identification** | **Description** | **Ontology** |
| --- | --- | --- |
| GO:0018335 | protein succinylation | biological_process |
| GO:0006104 | succinyl-CoA metabolic process | biological_process |
| GO:0042709 | succinate-CoA ligase complex | cellular_component |
| GO:0050161 | succinyl-CoA:oxalate CoA-transferase | molecular_function |
| GO:0033877 | succinyl-CoA:(R)-benzylsuccinate CoA-transferase activity | molecular_function |
| GO:0120226 | succinyl-CoA binding | molecular_function |
| GO:0004774 | succinate-CoA ligase activity | molecular_function |
| GO:0004778 | succinyl-CoA hydrolase activity | molecular_function |
| GO:0043961 | succinyl-CoA:(R)-citramalate CoA-transferase activity | molecular_function |
| GO:0008260 | succinyl-CoA:3-oxo-acid CoA-transferase activity | molecular_function |
| GO:0006105 | succinate metabolic process | biological_process |
| GO:0000104 | succinate dehydrogenase activity | molecular_function |
| GO:0015744 | succinate transport | biological_process |
