# Supplementary material for: Tandem mass tag-based quantitative proteomic analysis identification of succinylation related proteins in pathogenesis of thoracic aortic aneurysm and aortic dissection
Source: PeerJ. 2023 May 11;11:e15258. doi: 10.7717/peerj.15258 (PMC10183161; doi:10.7717/peerj.15258)
Supplement: Supplemental Information 4 [file peerj-11-15258-s004.docx]

**Table S3** Upregulated differentially expressed proteins in TAA and TAD patients compared with healthy controls

| **Protein**  **Symbol** | **Protein Name** | **Dissection.vs.Control Fold Change** | **Dissection.vs.Control P-value** | **Aneurysm.vs.Control Fold Change** | **Aneurysm.vs.Control P-value** |
| --- | --- | --- | --- | --- | --- |
| TGFB1 | Transforming growth factor beta | 1.264484033 | 0.00313752 | 1.31819663 | 0.002235215 |
| FN1 | Fibronectin 1 | 1.343547225 | 0.03297213 | 1.39670523 | 0.003578463 |
| PPP1R14B | Protein phosphatase 1, regulatory (Inhibitor) subunit 14B | 1.212334114 | 0.004854686 | 1.219042363 | 0.000770876 |
| CORO1A | Coronin | 1.481088342 | 0.014585897 | 1.298778454 | 0.04380421 |
| TIMM9 | Translocase of inner mitochondrial membrane 9 | 1.573245071 | 0.006935902 | 1.568427148 | 0.001882102 |
| CAMKK2 | Calcium/calmodulin-dependent protein kinase kinase 2, | 1.225591536 | 0.001887398 | 1.310598319 | 0.02066912 |
| MGLL | Monoglyceride lipase | 1.261451915 | 0.00048195 | 1.231502137 | 0.005612553 |
| HLA-DRA | HLA-DRA | 1.770420122 | 0.043629717 | 1.441433696 | 0.005677559 |
| TPM3 | Tropomyosin 3 isoform 3 | 1.382010211 | 0.000944743 | 1.286494129 | 0.00263052 |
| CD55 | Cromer blood group antigen | 1.362273328 | 0.01200271 | 1.296569781 | 0.002854968 |
| TPM4 | Tropomyosin alpha-4 chain | 1.354842089 | 0.008813238 | 1.566473232 | 2.71113E-05 |
| BST1 | Bone marrow stromal cell antigen 1 variant 2 | 1.645599116 | 0.002069822 | 1.848015424 | 0.000247731 |
| THBS2 | Thrombospondin-2 | 1.718869961 | 0.003828418 | 1.767085628 | 0.012048385 |
| HBB | Hemoglobin subunit beta | 1.922425891 | 0.021212088 | 1.679932715 | 0.003569018 |
| SEPTIN4 | Septin-4 | 1.203679274 | 0.017680078 | 1.331320521 | 0.002379901 |
| UCC1 | Ependymin related protein 1 | 1.490229775 | 0.000194139 | 1.820915421 | 0.000731695 |
| CP | CP protein | 1.7884487 | 0.003664248 | 1.473950855 | 0.026232488 |
| NUDT5 | ADP-sugar pyrophosphatase | 1.406833213 | 0.001251336 | 1.376736815 | 0.000625177 |
| SMCHD1 | Structural maintenance of chromosomes flexible hinge domain-containing protein 1 | 1.226667249 | 0.000339421 | 1.291929944 | 0.000311614 |
| FN1 | Fibronectin splice variant D | 2.332124128 | 1.75E-05 | 2.576769873 | 1.70879E-06 |
| CD55 | Complement decay-accelerating factor | 1.756669361 | 0.007804473 | 1.61212635 | 0.014648506 |
| COL6A3 | COL6A3 protein | 1.348207988 | 0.016486933 | 1.987308226 | 0.000319424 |
| CDKN2C | Cyclin-dependent kinase inhibitor 2C | 1.416478849 | 0.000253391 | 1.338185865 | 0.001267898 |
| S100A10 | S100 calcium binding protein A10 | 1.505856468 | 3.27E-05 | 1.646204548 | 5.13327E-05 |
| THY1 | Thy-1 membrane glycoprotein | 1.979497199 | 0.012410691 | 2.287101042 | 0.002993563 |
| PGM3 | Phosphoacetylglucosamine mutase | 1.307698535 | 0.003383671 | 1.279574637 | 0.000417967 |
| EBNA1BP2 | EBNA1 binding protein 2 | 1.226214043 | 0.018509903 | 1.235304942 | 0.000106552 |
| TPM3 | Tropomyosin alpha-3 chain | 1.551361671 | 0.005208098 | 1.586615419 | 0.000704182 |
| TPD52L1 | Tumor protein D53 | 1.340152045 | 0.002207748 | 1.735676832 | 5.63664E-05 |
| SLC16A3 | Monocarboxylate transporter 4 | 1.617120875 | 0.00125119 | 1.663003231 | 0.027810937 |
| RGS10 | Regulator of G-protein signaling 10 | 1.49962128 | 0.005159163 | 1.275090626 | 0.022631536 |
| ZNRD2 | Protein ZNRD2 | 1.22759776 | 0.001443795 | 1.219718971 | 0.000582641 |
| UGDH | UDP-glucose 6-dehydrogenase | 1.264217877 | 0.038595268 | 1.30619775 | 0.000933495 |
| FLNB | Filamin-B | 1.316406079 | 0.005268249 | 1.216940421 | 0.011836032 |
| HSBP1 | Heat shock factor-binding protein 1 | 1.255876829 | 0.000231253 | 1.320319948 | 0.000145784 |
| ALDH1A2 | Retinal dehydrogenase 2 | 1.967934041 | 0.001186038 | 1.631778532 | 0.001249136 |
| NDUFC2 | NADH dehydrogenase [ubiquinone] 1  subunit C2 | 1.230601679 | 0.00290898 | 1.245502384 | 0.000350695 |
| LDHA | L-lactate dehydrogenase A chain | 1.351717639 | 0.006290489 | 1.464984193 | 0.000206625 |
| HBD | Hemoglobin subunit delta | 2.012814671 | 0.01731155 | 1.725295623 | 0.001164447 |
| HPX | Hemopexin | 1.435106078 | 0.036756313 | 1.246887596 | 0.044944605 |
| A1BG | Alpha-1B-glycoprotein | 1.542734438 | 0.007934472 | 1.416821058 | 0.027206129 |
| SERPINA5 | Plasma serine protease inhibitor | 1.334944848 | 0.037221703 | 1.25476077 | 0.022765565 |
| CLEC3B | Tetranectin | 1.58821544 | 0.007946546 | 1.636265122 | 0.024618834 |
| SERPIND1 | Heparin cofactor 2 | 1.999983289 | 0.024787894 | 1.606948774 | 0.007538869 |
| PYGL | Glycogen phosphorylase, liver form | 1.258717641 | 0.000558641 | 1.201823383 | 0.000791047 |
| CTSD | Cathepsin D | 1.349937636 | 0.015387327 | 1.208672195 | 0.011096588 |
| ANXA3 | Annexin A3 | 1.576688593 | 0.008243376 | 1.943750009 | 0.013082689 |
| PLS3 | Plastin-3 | 1.279426923 | 0.024616593 | 1.340307256 | 0.00113235 |
| CD59 | CD59 glycoprotein | 1.555576064 | 0.004852285 | 1.518924238 | 0.001467615 |
| PML | Protein PML | 1.219984312 | 0.005093512 | 1.254749986 | 0.000803936 |
| MARCKS | Myristoylated alanine-rich C-kinase substrate | 2.292070101 | 0.000143471 | 2.374513596 | 2.60801E-05 |
| SERPINF1 | Pigment epithelium-derived factor | 1.301718308 | 0.01251407 | 1.214597005 | 0.025802057 |
| TAGLN2 | Transgelin-2 | 1.286835776 | 0.012537967 | 1.300314474 | 0.013940614 |
| RPL35 | 60S ribosomal protein L35 | 1.244870775 | 0.032139647 | 1.209159997 | 0.04829568 |
| CRIP1 | Cysteine-rich protein 1 | 1.954319828 | 0.001798852 | 2.456693242 | 0.000433454 |
| PGD | 6-phosphogluconate dehydrogenase, decarboxylating | 1.316721053 | 0.001506047 | 1.220157705 | 0.000413702 |
| ARHGDIB | Rho GDP-dissociation inhibitor 2 | 1.384402244 | 0.023073328 | 1.45569157 | 0.001636212 |
| STAT2 | Signal transducer and activator of  transcription 2 | 1.271932 | 0.014615651 | 1.366162428 | 0.000909672 |
| CRIP2 | Cysteine-rich protein 2 | 1.315630151 | 0.012556367 | 1.423517876 | 0.000748945 |
| ITGA1 | Integrin alpha-1 | 1.425620014 | 0.000132507 | 1.386148135 | 0.000846642 |
| TPI1 | Triosephosphate isomerase | 1.251117508 | 0.000526503 | 1.246374826 | 0.003631808 |
| GNG5 | Guanine nucleotide-binding protein G(I)/G(S)/G(O) subunit gamma-5 | 1.84250493 | 0.002579107 | 1.567852755 | 0.006966015 |
| TPM4 | Tropomyosin alpha-4 chain | 1.457085426 | 0.004568027 | 1.654974822 | 3.1105E-05 |
| COL16A1 | Collagen alpha-1(XVI) chain | 1.717686289 | 0.008243051 | 1.583379231 | 0.007264746 |
| DPYD | Dihydropyrimidine dehydrogenase [NADP(+)] | 1.801305078 | 3.32E-06 | 1.892741308 | 2.50986E-06 |
| MAPRE2 | Microtubule-associated protein RP/EB family member 2 | 1.236543068 | 0.003195705 | 1.272897846 | 0.004496668 |
| FSCN1 | Fascin actin-bundling protein 1 | 1.265131593 | 0.029644501 | 1.582316643 | 0.000200918 |
| FNDC3B | Fibronectin type III domain-containing  protein 3B | 1.362601405 | 0.002853247 | 1.251387391 | 0.001027891 |
| TRG14 | Transformation-related protein 14 | 1.290417497 | 0.000231396 | 1.211680198 | 0.009234405 |
| PLAC9 | Placenta-specific protein 9 | 1.666186603 | 0.000131832 | 1.556614704 | 0.001289464 |
| AIF1 | Allograft inflammatory factor 1 | 2.357944099 | 0.000557493 | 1.647374673 | 0.000442815 |
| SNCG | Gamma-synuclein | 2.045010252 | 0.000249487 | 3.006107101 | 0.000578718 |
| SFRP4 | Secreted frizzled-related protein 4 | 1.680799142 | 0.002718346 | 3.532412862 | 7.55696E-06 |
| DKFZp686K03196 | Uncharacterized protein | 1.80756769 | 0.04001363 | 2.251767526 | 0.006750468 |
| LHFPL2 | LHFPL tetraspan subfamily member 2 protein | 1.396879753 | 0.00751047 | 1.456966179 | 0.000557912 |
| IKBIP | Inhibitor of nuclear factor kappa-B kinase-interacting protein | 1.210363425 | 0.011259951 | 1.403616791 | 0.005959402 |
| AHNAK2 | Protein AHNAK2 | 1.547583659 | 5.53E-05 | 1.918837603 | 0.001944709 |
| FMNL3 | Formin-like protein 3 | 1.274753082 | 0.003453718 | 1.311048197 | 0.006206488 |
| OXR1 | Oxidation resistance protein 1 | 1.215036449 | 0.018237899 | 1.260128672 | 0.000795025 |
| TSTD1 | Thiosulfate:glutathione sulfurtransferase | 1.260208998 | 0.012090067 | 1.520316427 | 0.002700258 |
| SH3KBP1 | SH3 domain-containing kinase-binding protein 1 | 1.28730227 | 0.00024007 | 1.234754043 | 0.002169447 |
| S100A13 | Protein S100-A13 | 1.373975642 | 0.003590138 | 1.424227037 | 0.000253979 |
| C1QTNF5 | Complement C1q tumor necrosis factor-related protein 5 | 1.926105213 | 0.000761741 | 1.506167813 | 0.003194787 |
| UACA | Uveal autoantigen with coiled-coil domains and ankyrin repeats | 1.203574118 | 0.003306224 | 1.335280465 | 4.02758E-05 |
| WWTR1 | WW domain-containing transcription regulator protein 1 | 1.547547811 | 0.001083352 | 1.475886893 | 1.87795E-05 |
| CRISPLD1 | Cysteine-rich secretory protein LCCL domain-containing 1 | 1.30931218 | 0.02568253 | 1.216735595 | 0.020352586 |
| COPZ2 | Coatomer subunit zeta-2 | 1.296247386 | 0.003310162 | 1.536844251 | 0.00015289 |
| DPP7 | Dipeptidyl peptidase 2 | 1.373150101 | 0.000193462 | 1.296879755 | 0.00041261 |
| EVL | Ena/VASP-like protein | 1.218683552 | 0.008032424 | 1.208378526 | 0.007944883 |
| LAMTOR2 | Ragulator complex protein LAMTOR2 | 1.306661742 | 2.21E-05 | 1.204428866 | 0.000826497 |
| HEL-117 | Ubiquitin carboxyl-terminal hydrolase | 1.797505008 | 0.004088142 | 1.858401773 | 0.001824542 |
| HEL-S-11 | Carbonic anhydrase I | 1.929674047 | 0.028457225 | 1.730448574 | 0.008814509 |
| HEL-S-51 | Epididymis secretory protein Li 51 | 1.402768365 | 0.041669225 | 1.23165669 | 0.047451464 |
