# Supplementary material for: Tandem mass tag-based quantitative proteomic analysis identification of succinylation related proteins in pathogenesis of thoracic aortic aneurysm and aortic dissection
Source: PeerJ. 2023 May 11;11:e15258. doi: 10.7717/peerj.15258 (PMC10183161; doi:10.7717/peerj.15258)
Supplement: Supplemental Information 5 [file peerj-11-15258-s005.docx]

**Table S4** Downregulated differentially expressed proteins in TAA and TAD patients compared with healthy controls

| **Protein**  **Symbol** | **Protein**  **Name** | **Dissection.vs.Control Fold Change** | **Dissection.vs.Control P-value** | **Aneurysm.vs.Control Fold Change** | **Aneurysm.vs.Control P-value** |
| --- | --- | --- | --- | --- | --- |
| PRAF2 | PRA1 family protein | 0.661498303 | 8.10E-06 | 0.726044 | 0.000341 |
| XPC | Xeroderma pigmentosum, complementation group C | 0.648373495 | 0.00016004 | 0.564259 | 6.35E-05 |
| DLAT | Acetyltransferase component of pyruvate dehydrogenase complex | 0.749070222 | 4.26E-05 | 0.798931 | 0.000645 |
| PLEKHC1 | Pleckstrin homology domain containing, family C (With FERM domain) member 1 | 0.816895341 | 0.00467831 | 0.815097 | 0.006674 |
| BRP44 | Mitochondrial pyruvate carrier | 0.764155452 | 0.00047172 | 0.730887 | 0.006984 |
| UBE2D4 | Ubiquitin-conjugating enzyme E2D 4 (Putative) | 0.830351382 | 0.00787959 | 0.784314 | 0.000449 |
| CSPG2 | Chondroitin sulfate proteoglycan 2 (Versican) | 0.645228523 | 0.01644467 | 0.603786 | 0.000581 |
| MTM1 | Myotubularin 1 | 0.780622125 | 0.00036542 | 0.731525 | 0.000442 |
| CD151 | Tetraspanin | 0.734499326 | 0.00276247 | 0.661855 | 3.78E-05 |
| SMTN | Smoothelin | 0.626073178 | 6.59E-05 | 0.700796 | 6.19E-06 |
| TMEM120A | Transmembrane protein 120A | 0.70952854 | 0.00022183 | 0.728997 | 0.00052 |
| LU | Lutheran blood group glycoprotein | 0.729882817 | 0.00476418 | 0.487053 | 1.35E-06 |
| PON2 | Paraoxonase 2 | 0.742406451 | 0.00799116 | 0.670887 | 0.001244 |
| ARAF | V-raf murine sarcoma 3611 viral oncogene-like protein isoform 2 | 0.748711992 | 0.00089898 | 0.710036 | 0.000198 |
| ACADSB | Acyl-CoA dehydrogenase short/branched chain isoform 1 | 0.730806801 | 0.00731098 | 0.727792 | 0.006101 |
| LDB3 | LIM domain binding 3 isoform 1 | 0.668577322 | 0.00157426 | 0.770969 | 0.014177 |
| DCP1A | DCP1 decapping enzyme-like protein A isoform 1 | 0.827437756 | 0.03628934 | 0.780477 | 0.002455 |
| GSN | Gelsolin | 0.803789423 | 0.02752913 | 0.70791 | 0.002274 |
| ANO1 | Anoctamin | 0.826331038 | 0.04851377 | 0.684277 | 6.76E-05 |
| ARHGAP10 | ARHGAP10 | 0.783563754 | 0.00088184 | 0.777705 | 0.00011 |
| ALDH2 | Aldehyde dehydrogenase 2 family (Mitochondrial) | 0.797690369 | 0.00396076 | 0.814021 | 0.015538 |
| ADIG | Adiponectin G | 0.699488883 | 0.0231917 | 0.588594 | 0.004062 |
| SLMAP | Sarcolemmal membrane-associated protein | 0.552162913 | 0.00012983 | 0.676981 | 0.001016 |
| ACAT1 | Acetyl-CoA acetyltransferase 1 | 0.797862281 | 0.00065523 | 0.815367 | 0.000365 |
| RAB27A | RAB27A, member RAS oncogene family | 0.803644058 | 0.00434912 | 0.755392 | 0.000515 |
| PHKA1 | Phosphorylase b kinase regulatory subunit | 0.800882499 | 0.01449562 | 0.770233 | 0.0073 |
| RBPMS | HCG2043421 | 0.829852593 | 0.00215137 | 0.800165 | 0.0002 |
| HDAC1 | Histone deacetylase | 0.777277901 | 0.00025686 | 0.739304 | 4.94E-05 |
| COMT | Catechol O-methyltransferase | 0.809827416 | 0.00739424 | 0.811219 | 0.011639 |
| SYNPO2 | Synaptopodin 2 | 0.780819891 | 0.01918584 | 0.74455 | 0.005785 |
| NES | Nestin | 0.694181261 | 0.00448743 | 0.601984 | 0.000472 |
| XPO4 | Exportin-4 | 0.756407021 | 0.00076316 | 0.65454 | 0.000183 |
| HAPLN3 | Hyaluronan and proteoglycan link protein 3 | 0.605701778 | 0.00024398 | 0.689137 | 0.015831 |
| GULP1 | PTB domain-containing engulfment adapter protein 1 | 0.532640737 | 0.00023223 | 0.502593 | 0.000112 |
| MFAP4 | Microfibril-associated glycoprotein 4 | 0.723792903 | 0.00653443 | 0.519812 | 1.49E-05 |
| KPNA4 | Importin subunit alpha-3 | 0.809211577 | 0.00241254 | 0.761622 | 5.67E-05 |
| LAMA5 | Laminin subunit alpha-5 | 0.780443094 | 0.00329135 | 0.503086 | 2.88E-07 |
| CYB5B | Cytochrome b5 type B | 0.778378539 | 0.00028628 | 0.675955 | 6.58E-06 |
| PROM1 | Prominin-1 | 0.746395572 | 0.00010318 | 0.802645 | 0.001862 |
| ZMPSTE24 | CAAX prenyl protease 1 homolog | 0.821797282 | 0.00577085 | 0.763643 | 0.00033 |
| DYSF | Dysferlin | 0.702809859 | 0.0046016 | 0.623695 | 0.001266 |
| NTN1 | Netrin-1 | 0.478446403 | 0.00438301 | 0.375196 | 0.000645 |
| TOMM40 | Mitochondrial import receptor subunit  TOM40 homolog | 0.810791831 | 0.00360281 | 0.713767 | 4.3E-05 |
| CRYAB | Alpha-crystallin B chain | 0.807791084 | 0.04662315 | 0.697762 | 0.00062 |
| IGF1R | Insulin-like growth factor 1 receptor | 0.73845047 | 0.00035491 | 0.760089 | 0.000283 |
| SOD3 | Extracellular superoxide dismutase [Cu-Zn] | 0.742088571 | 0.01986706 | 0.696526 | 0.004048 |
| LAMC1 | Laminin subunit gamma-1 | 0.696329155 | 0.00024704 | 0.52179 | 1.99E-06 |
| DMD | Dystrophin | 0.782448466 | 0.00305425 | 0.757029 | 0.001786 |
| NID1 | Nidogen-1 | 0.81129271 | 0.00289191 | 0.684974 | 9.28E-05 |
| NPR1 | Atrial natriuretic peptide receptor 1 | 0.795154783 | 0.01830416 | 0.7674 | 0.008713 |
| LAMA2 | Laminin subunit alpha-2 | 0.795583766 | 0.03389554 | 0.617416 | 0.003831 |
| IGFBP5 | Insulin-like growth factor-binding protein 5 | 0.703701326 | 3.89E-05 | 0.787662 | 0.004121 |
| ALDH4A1 | Delta-1-pyrroline-5-carboxylate dehydrogenase, mitochondrial | 0.830394304 | 0.00245028 | 0.812435 | 0.001181 |
| GCHFR | GTP cyclohydrolase 1 feedback regulatory protein | 0.672376894 | 0.00335793 | 0.775381 | 0.011148 |
| AXL | Tyrosine-protein kinase receptor UFO | 0.652894297 | 0.0050213 | 0.749886 | 0.020706 |
| CAPZB | F-actin-capping protein subunit beta | 0.587338194 | 0.00120936 | 0.765927 | 0.047329 |
| ENTPD1 | Ectonucleoside triphosphate diphosphohydrolase 1 | 0.804313528 | 0.02704367 | 0.652336 | 0.001003 |
| THOP1 | Thimet oligopeptidase | 0.824034227 | 0.0058276 | 0.825826 | 0.034743 |
| LAMB2 | Laminin subunit beta-2 | 0.730032902 | 0.00163087 | 0.469679 | 5.47E-06 |
| OXCT1 | Succinyl-CoA:3-ketoacid coenzyme A transferase 1 | 0.668914589 | 0.00114815 | 0.752407 | 0.004444 |
| EPPK1 | Epiplakin | 0.712664498 | 0.02351865 | 0.65374 | 0.008317 |
| RRAS2 | Ras-related protein R-Ras2 | 0.795955829 | 0.01241492 | 0.750476 | 0.00082 |
| RHOB | Rho-related GTP-binding protein RhoB | 0.707296105 | 0.00100772 | 0.541483 | 1.54E-05 |
| RAP1A | Ras-related protein Rap-1A | 0.795495636 | 0.00139312 | 0.689726 | 5.07E-05 |
| ADARB1 | Double-stranded RNA-specific editase 1 | 0.791106847 | 0.00133078 | 0.652238 | 1.4E-05 |
| MRPS36 | 28S ribosomal protein S36, mitochondrial | 0.832128935 | 0.00101381 | 0.812208 | 5.28E-05 |
| HTRA3 | Serine protease HTRA3 | 0.758237797 | 0.0012104 | 0.663739 | 5.74E-05 |
| PTP4A2 | Protein tyrosine phosphatase type IVA 2 | 0.605373305 | 0.04211692 | 0.502672 | 0.024138 |
| PTPRS | Receptor-type tyrosine-protein phosphatase S | 0.811482596 | 0.00860342 | 0.761117 | 9.06E-05 |
| ITGA9 | Integrin alpha-9 | 0.78957843 | 0.04285272 | 0.742308 | 0.015896 |
| FLNC | Filamin-C | 0.652388774 | 0.00233034 | 0.626967 | 0.000223 |
| SPARCL1 | SPARC-like protein 1 | 0.578266223 | 0.00668455 | 0.415373 | 0.00044 |
| LPIN1 | Phosphatidate phosphatase LPIN1 | 0.815078466 | 0.01782394 | 0.721427 | 0.000775 |
| PGM5 | Phosphoglucomutase-like protein 5 | 0.809442199 | 0.00394511 | 0.826835 | 0.001498 |
| ALG11 | GDP-Man:Man(3)GlcNAc(2)-PP-Dol alpha-1,2-mannosyltransferase | 0.790350409 | 0.00070694 | 0.685615 | 0.000105 |
| FILIP1L | Filamin A-interacting protein 1-like | 0.548821668 | 0.00085449 | 0.700286 | 0.006226 |
| CMPK2 | UMP-CMP kinase 2, mitochondrial | 0.584573181 | 0.00068677 | 0.749702 | 0.015605 |
| NHSL2 | NHS-like protein 2 | 0.575468685 | 0.00162638 | 0.56437 | 0.001445 |
| NXN | Nucleoredoxin | 0.699666397 | 0.00022148 | 0.800693 | 0.002186 |
| PGRMC1 | PGRMC1 protein | 0.819113598 | 0.00638329 | 0.763666 | 0.002409 |
| DKFZp686N08224 | Uncharacterized protein DKFZp686N08224 | 0.612014842 | 0.01124274 | 0.637069 | 0.01214 |
| THOC6 | THO complex subunit 6 homolog | 0.710149442 | 0.00036948 | 0.804943 | 0.01056 |
| SBSPON | Somatomedin-B and thrombospondin type-1 domain-containing protein | 0.739538366 | 0.00088008 | 0.473643 | 1.11E-05 |
| CNTN4 | Contactin-4 | 0.716352923 | 0.0191663 | 0.725086 | 0.004227 |
| RHOT1 | Mitochondrial Rho GTPase 1 | 0.775157898 | 0.00012972 | 0.822747 | 0.000848 |
| FIBIN | Fin bud initiation factor homolog | 0.784525202 | 0.00530443 | 0.659116 | 0.001572 |
| SCFD2 | Sec1 family domain-containing protein 2 | 0.815965635 | 0.00455652 | 0.748288 | 0.002457 |
| TAF15 | TATA-binding protein-associated factor 2N | 0.830106919 | 0.02706906 | 0.827851 | 0.005972 |
| IGSF8 | Immunoglobulin superfamily member 8 | 0.808128047 | 0.03531894 | 0.81876 | 0.008551 |
| RERG | Ras-related and estrogen-regulated growth inhibitor | 0.772407394 | 0.00883113 | 0.741717 | 0.00131 |
| ESAM | Endothelial cell-selective adhesion molecule | 0.763249115 | 0.02986472 | 0.765825 | 0.034883 |
| RHBDF1 | Inactive rhomboid protein 1 | 0.816050731 | 0.01486467 | 0.736243 | 0.002483 |
| TRIM47 | E3 ubiquitin-protein ligase TRIM47 | 0.799093364 | 0.01131183 | 0.722701 | 0.000962 |
| LURAP1 | Leucine rich adaptor protein 1 | 0.48479058 | 0.00095207 | 0.491526 | 0.000595 |
| MGST2 | Microsomal glutathione S-transferase 2 | 0.750032163 | 0.00030318 | 0.741954 | 0.000586 |
| JPH2 | Junctophilin-2 | 0.715399555 | 0.00013615 | 0.824277 | 0.002236 |
| SORBS1 | Sorbin and SH3 domain-containing protein 1 | 0.678400553 | 0.00218264 | 0.693198 | 0.0058 |
| UBE2O | (E3-independent) E2 ubiquitin-conjugating enzyme | 0.827782935 | 0.00490205 | 0.734383 | 0.000279 |
| MFF | Mitochondrial fission factor | 0.784373747 | 2.45E-05 | 0.815811 | 1.23E-05 |
| SMOC2 | SPARC-related modular calcium-binding protein 2 | 0.755033833 | 0.021109 | 0.802961 | 0.039165 |
| MINDY3 | Ubiquitin carboxyl-terminal hydrolase MINDY-3 | 0.799224076 | 0.00294146 | 0.799695 | 0.000829 |
| SDR39U1 | Epimerase family protein SDR39U1 | 0.782440247 | 0.00109588 | 0.748133 | 0.000588 |
| PARVA | Alpha-parvin | 0.707436106 | 6.14E-05 | 0.822829 | 0.002847 |
| DNAJC11 | DnaJ homolog subfamily C member 11 | 0.79032803 | 0.00068851 | 0.785868 | 1.46E-05 |
