# Supplementary material for: Tandem mass tag-based quantitative proteomic analysis identification of succinylation related proteins in pathogenesis of thoracic aortic aneurysm and aortic dissection
Source: PeerJ. 2023 May 11;11:e15258. doi: 10.7717/peerj.15258 (PMC10183161; doi:10.7717/peerj.15258)
Supplement: Supplemental Information 8 [file peerj-11-15258-s008.docx]

**Figure 1A Succinylation**

**
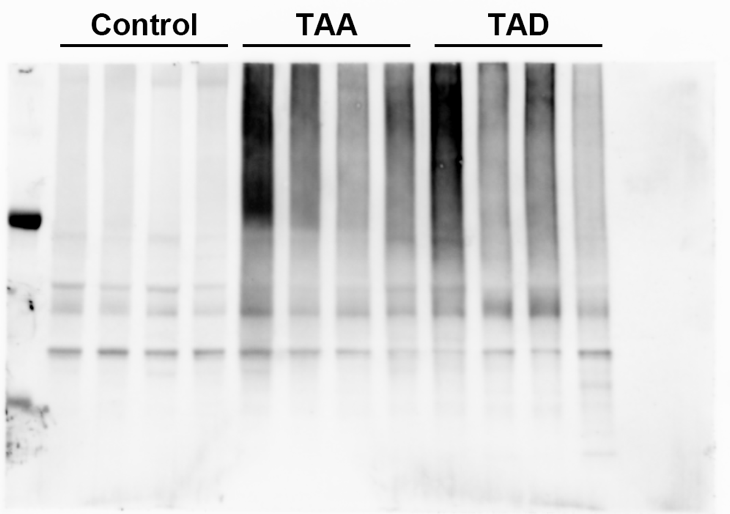
**

**Figure 1B Ubiquitination**

**
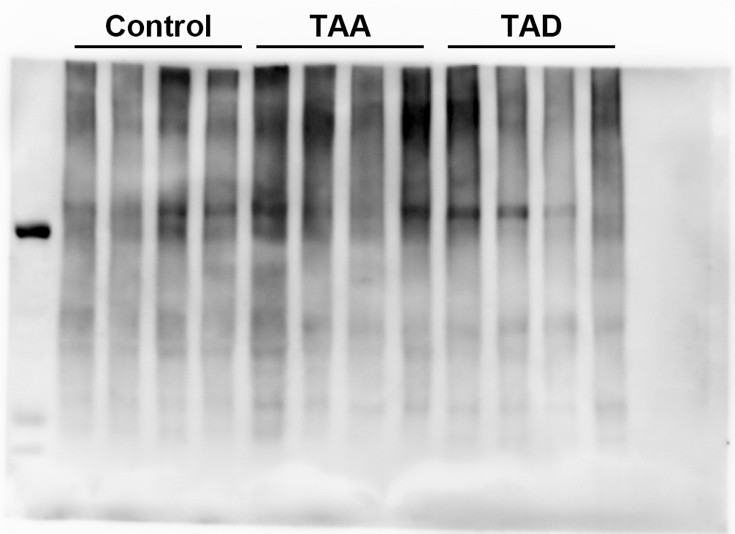
**

**Figure 1C Malonylation**

**
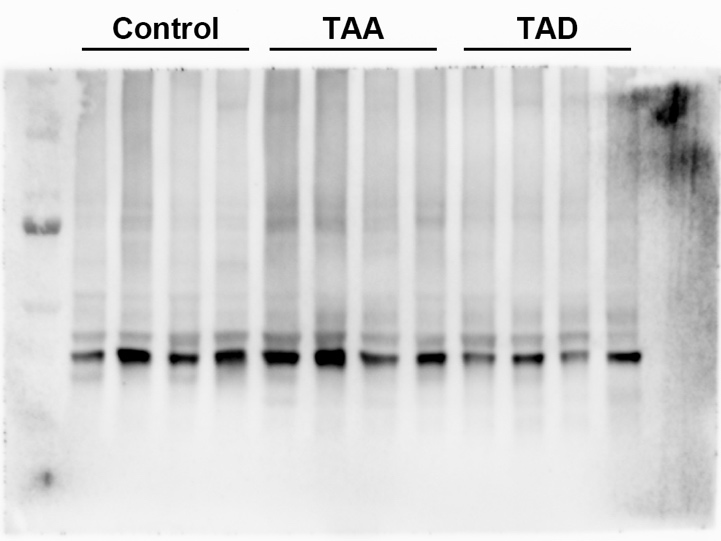
**

**Figure 1D Acetylation**

**
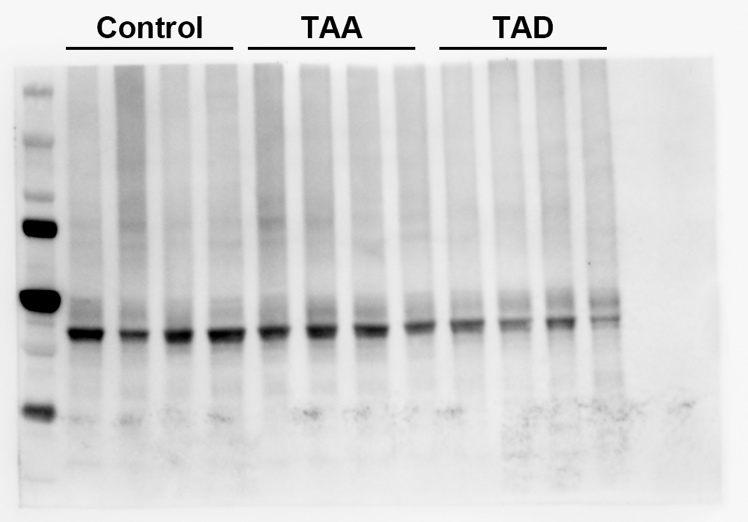
**

**Figure 1E Lactylation**

**
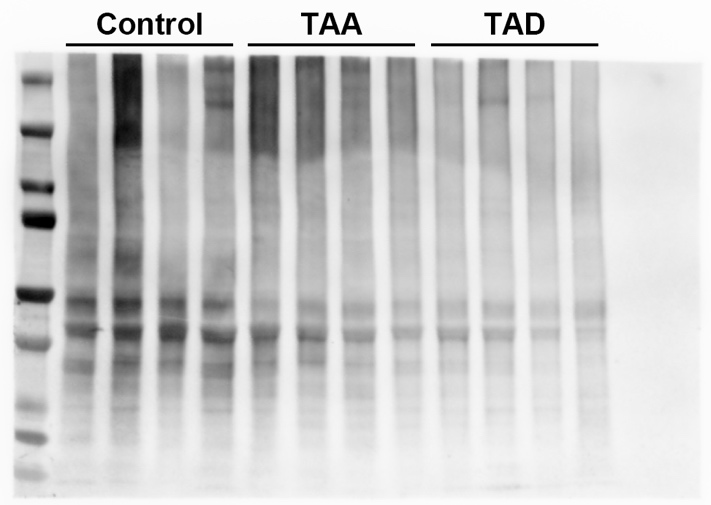
**

**Figure 1F Coommassie blue staining**

**
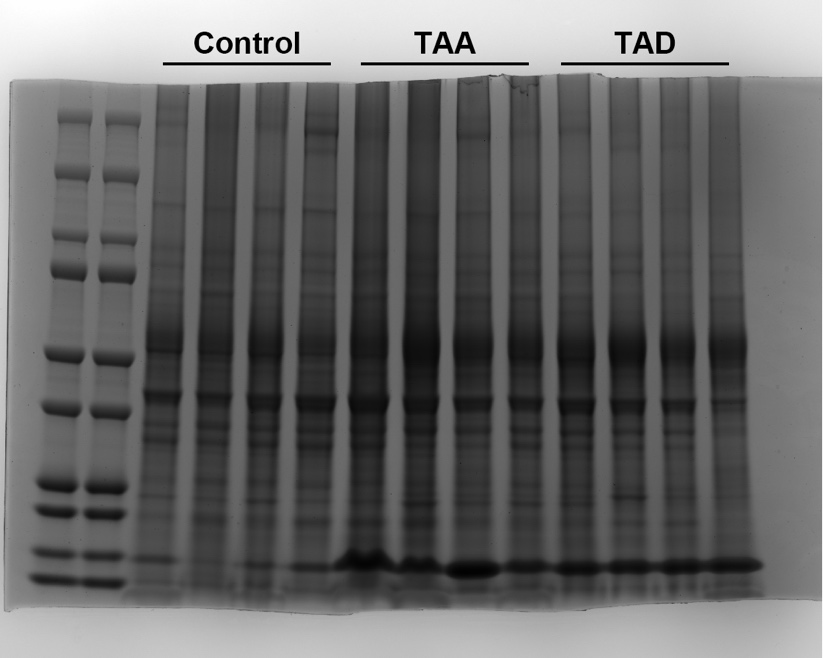
**

**Figure 5B**

**OXCT1**

**
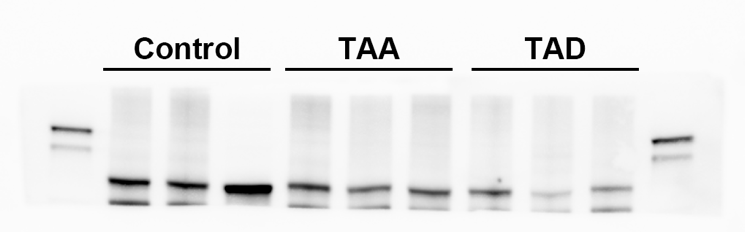
**

**GAPDH**

**
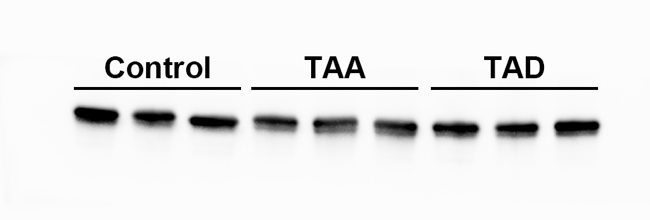
**

**Figure S1**

**OXCT1**

**
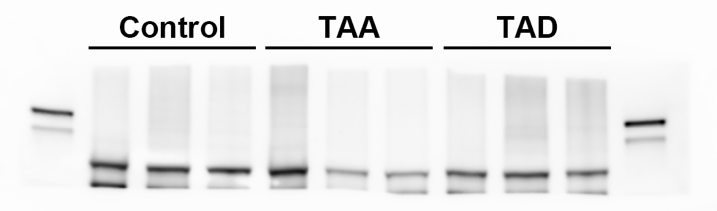
**

**GAPDH**

**
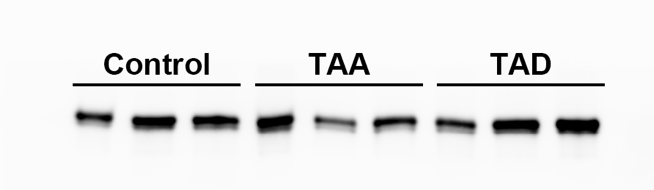
**
